# Early thromboelastography in acute traumatic coagulopathy: an observational study focusing on pre-hospital trauma care

**DOI:** 10.1007/s00068-020-01493-z

**Published:** 2020-09-14

**Authors:** Alessandra Spasiano, Cristina Barbarino, Anna Marangone, Daniele Orso, Giulio Trillò, Roberta Giacomello, Tiziana Bove, Giorgio Della Rocca

**Affiliations:** 1 Anesthesiology and Intensive Care Medicine, Department of Medicine, University of Udine, ASUFC Udine, P.le Santa Maria della Misericordia 15, 33100 Udine, Italy; 2 HEMS Division, Department of Anesthesia and Intensive Care Medicine, ASUFC Udine, 33100 Udine, Italy; 3 Department of Laboratory Medicine, Institute of Clinical Pathology, University of Udine, ASUFC Udine, 33100 Udine, Italy

**Keywords:** Trauma-associated coagulopathy, Thromboelastography, Fibrinolysis, Trauma, Coagulation disorder, TEG

## Abstract

**Background:**

Major brain injury and uncontrolled blood loss remain the primary causes of early trauma-related mortality. One-quarter to one-third of trauma patients exhibit trauma-induced coagulopathy (TIC). Thromboelastometry (ROTEM) and thrombelastography (TEG) are valuable alternatives to standard coagulation testing, providing a more comprehensive overview of the coagulation process.

**Purpose:**

Evaluating thromboelastographic profile, the incidence of fibrinolysis (defined as Ly30 > 3%) in severe trauma patients, and factors influencing pathological coagulation pattern.

**Methods:**

Prospective observational 2 years cohort study on severe trauma patients assisted by Helicopter Emergency Medical System (HEMS) and Level 1 Trauma Center, in a tertiary referral University Hospital.

**Results:**

Eighty three patients were enrolled, mean NISS (new injury severity score) 36 (± 13). Mean *R* value decreased from 7.25 (± 2.6) to 6.19 (± 2.5) min (*p* < 0.03); 48 (60%) patients had a reduction in *R* from *T*_0_ to *T*_1_. In NISS 25–40 and NISS > 40 groups, changes in *R* value increased their significance (*p* = 0.04 and *p* < 0.03, respectively). Pathological TEG was found in 71 (88.8%) patients at *T*_0_ and 74 (92.5%) at *T*_1_. Hypercoagulation was present in 57 (71.3%) patients at *T*_0,_ and in 66(82.5%) at *T*_1_. 9 (11.3%) patients had hyperfibrinolysis at *T*_0_, 7 (8.8%) patients at *T*_1_. Prevalence of StO_2_ < 75% at *T*_0_ was greater in patients whose TEG worsened (7 patients, 46.7%) against whose TEG remained stable or improved (8 patients, 17.4%) from *T*_0_ to *T*_1_ (*p* = 0.02). 48 (57.8%) patients received < 1000 mL of fluids, while 35 (42.2%) received ≥ 1000 mL. The first group had fewer patients with hypercoagulation (20, 41.6%) than the second (6, 17.6%) at *T*_1_ (*p* < 0.03). No differences were found for same TEG pattern at *T*_0_, nor other TEG pattern.

**Conclusion:**

Our population is representative of a non-hemorrhagic severe injury subgroup. Almost all of our trauma population had coagulation abnormalities immediately after the trauma; pro-coagulant changes were the most represented regardless of the severity of injury. NISS appears to affect only *R* parameter on TEG. Hyperfibrinolysis has been found in a low percentage of patients. Hypoperfusion parameters do not help to identify patients with ongoing coagulation impairment. Small volume resuscitation and mild hypotermia does not affect coagulation, at least in the early post-traumatic phase.

**Electronic supplementary material:**

The online version of this article (10.1007/s00068-020-01493-z) contains supplementary material, which is available to authorized users.

## Introduction

Major brain injury and uncontrolled blood loss remain the primary causes of early trauma-related mortality [1–3]. One-quarter to one-third of trauma patients exhibit trauma-induced coagulopathy (TIC) [4, 5], which is associated with increased rates of massive transfusion (MT) and multiple organ failure (MOF), prolonged intensive care unit (ICU) and hospital length-of-stay, and a four-fold increase in mortality [4]. Most patients with coagulopathy have uncontrolled bleeding as well, and early diagnosis of the underlying coagulation disorder is paramount for effective treatment.

The pathogenesis of severe post-traumatic coagulopathy is complex and multifactorial. It involves factor-related primary to the trauma itself and, later, to medical treatment provided [6, 7]. Coagulopathy secondary to the extravasation of tissue factor and hypoperfusion is associated with a poor outcome such as MOF and death [8]. TIC—which differs from disseminated intravascular coagulation—is a result of enhanced activity of plasma coagulation during the initial phase. TIC is characterized by early-onset prolonged prothrombin time and activated partial thromboplastin time and a relative sparing of platelets and fibrinogen [9].

Viscoelastic hemostatic assays (VHA)—such as thromboelastometry (ROTEM) and thromboelastography (TEG)—are valuable alternatives to standard coagulation testing, providing a more comprehensive overview of the coagulation process [10, 11]. In contrast to most standard coagulation measurements, TEG can be used as a point-of-care method. VHAs permit goal-directed therapy for TIC [12].

Different studies have described an early post-traumatic coagulopathy in 24–36% of trauma patients upon admission to the emergency department [5, 13]. This early coagulopathy was a marker of injury severity and was related to mortality. More recently, TIC was found in a percentage from 8 to 14% of trauma patients, but a very different mode to diagnose it was highlighted [14–16]. Furthermore, up to date, there are only two prospective studies evaluating the initial coagulation status with VHA [17] and with VHA plus traditional analysis [15], on pre-hospital setting and at hospital admission, respectively. The hypothesis was that coagulation abnormalities became apparent very early, already on the accident site.

The purpose of this prospective observational cohort study is to describe the on-scene and on-hospital-arrival coagulation profile with a point-of-care TEG to detect early coagulation modification and fibrinolysis (Lys30 > 3%) in severe trauma patients.

## Materials and methods

Our hospital research ethics committee approved this single-center, prospective, observational study (codice CE). Because most of these patients would be unconscious, and the next of kin was not readily available, the requirement for informed consent, under the provisions of the ethics committee and because the observational nature of the study, was waived.

We enrolled in any severe trauma adult (> 18 years) patient rescued by our HEMS crew from December 2014 to December 2016. Severe trauma was defined as a new injury severity score (NISS) of more than 15. We excluded patients undergoing anticoagulant therapy before the trauma.

A blood count sample, a blood sample in citrate (for TEG and coagulation tests), and arterial blood gas (ABG) analysis were collected for each patient on the trauma scene (*T*_0_) and at hospital arrival (*T*_1_). *T*_0_ and *T*_1_ refers to time interval between activation of the emergency response system and, respectively, the arrival on trauma scene or hospital admission.

For each patient, we registered: demographic characteristics, type of trauma, trauma dynamic, vital parameters (non-invasive blood pressure (NIBP), heart rate (HR), respiratory rate (RR), oxygen saturation (SpO_2_), tissue oxygen saturation (StO_2_; InSpectra Model 325; Hutchinson Thecnology Inc., Hutchinson, MN, USA), temperature (°C) at *T*_0_ and *T*_1_, tranexamic acid and fluid administration, blood components transfused in first 24 h, NISS.

For TEG analysis, a Computerized Thromboelastograph Coagulation Analyzer^®^ (TEG model 5000, Haemoscope Corporation, Niles, IL, USA) was used. The instrument had quality control performed daily, and the tests performed were following manufacturer specifications. All analyses were performed by staff trained in their use with regular assessments on their ability to perform the tests. 340 µL of blood added with 20 µL of CaCl_2_ (0.2 M) was pipetted into a TEG cup pre-warmed to 37 °C. The following TEG variables were assessed: *R* time (reaction time; s), *K* (kinetics; s), angle (alpha angle, which means the slope of line between *R* and *K*; grades), MA (maximum amplitude; mm), *G* (a log-derivation of the MA, representing clot strenght; dyne/s), LY30 (lysis at 30 min, which means percentage decrease in amplitude at 30 min post-MA; %).

We collected Hematocrit (Htc, %), Hemoglobin (Hb, g dL^−1^), Platelet (PLT, *n*° × 103 μL^−1^) (Beckman Coulter Unicel^®^DxH 800 Coulter^®^, Miami, FL, USA) from blood count sample and Base Excess (BE in mmol L^−1^) from ABG. Coagulation laboratory tests Prothrombin Time (PT) (Recombiplastin 2G^®^, Instrumentation Laboratory Co, Orangeburg, USA), International Normalized Ratio (INR), Partial Thromboplastin Time (PTT) (APTT-SP^®^, Instrumentation Laboratory Co, Orangeburg, USA) and fibrinogen (FBN) (Q.F.A.^®^, ACL TOP 700, Instrumentation Laboratory Co, Orangeburg, USA) were obtained. Trauma-induced coagulopathy (TIC) was defined as INR > 1.5 [18]. Pathological TEG was defined as a TEG in which at least one parameter (between *R* time, MA and *G*) was out of normality range. Normality range: *R* time 9–27 s, MA 44–64 mm, *G* 3.6–8.5 dyne/s. Hypocoagulation was defined as at least 2 of these criteria: *R* > 27, MA < 44 or *G* < 3.6. Hypercoagulation was defined as at least two of these criteria: *R* < 9, MA > 64 or *G* > 8.5.

### Statistical analysis

Data are presented as the number of patients and percentage or as mean ± standard deviation and range (minimum and maximum). The groups were compared with the Mann–Whitney test. A *p* value ≤ 0.05 was considered statistically significant. All statistical analyses were performed using the R-CRAN project ver. 3.5.2.

## Results

We enrolled 94 trauma patients rescued by HEMS. Eleven patients were excluded: six because of non-traumatic emergencies, four because they presented a NISS < 15, and one patient for the absence of TEG data. We finally included 83 patients in the statistical analysis: demographic parameters, NISS, blood sample times and mechanism of injury are shown in Table 1. Vital and laboratory parameters are reported in Tables 2 and 3.

**Table 1:** Demographic parameters, NISS, blood sample times and mechanism of injury

No. of patients	83
M/F	61 (73.5%)/22 (26.5%)
Age (years)	54 ± 19; 18–93
NISS	36 ± 13; 16–75
Time of first blood sample (*T*_0_) (min)	22 ± 7; 9–52
Time of second blood sample (*T*_1_) (min)	55 ± 19; 23–127

Trauma cause	No. pts (%)
Motorcycle	22 (26.5)
Car	19 (22.9)
Bicycle	8 (9.6)
Run over by car/motorcycle	4 (4.8)
Crush	2 (2.4)
Fall from high	19 (22.9)
Other	9 (10.9)

Data are presented as No. of patients (No. pts) and percentage or as mean ± SD and range (minimum and maximum)

*M* male, *F* female, *NISS* new injury severity score

**Table 2:** Vital parameters (mean ± SD) and patients with hypoperfusion indices (No. of patients and percentage) at *T*_0_ and *T*_1_

	*T*_0_	*T*_1_	*p* value
sAP (mmHg)	121 ± 36	125 ± 27	0.45
HR (bpm)	91 ± 25	89 ± 19	0.29
*T* (°C)	36 ± 1.7	36 ± 1.6	0.06
SpO_2_ (%)	94 ± 6	98 ± 3	**0.001**
StO_2_ (%)	79 ± 10	82 ± 9	**0.003**
No. pts with sAP < 90 mmHg	14 (16.9%)	5 (6%)	**0.028**
No. pts with BE ≤ − 6 mmol L^−1^	13 (15.7%)	16 (19.3%)	0.53
No. pts with StO_2_ < 75%	16 (19.3%)	7 (8.4%)	0.04
No. pts with sAP < 90 mmHg + StO_2_ < 75%	3 (3.6%)	0	–

*p* values < 0.05 are in bold

*sAP* systolic arterial pressure, *HR* heart rate, *T* temperature, *SpO*_*2*_ peripheral oxygen saturation, *StO*_*2*_ tissue oxygen saturation, *BE* base excess

**Table 3:** Laboratory parameters and coagulation parameters (mean ± SD) at *T*_0_ and *T*_1_

	*T*_0_	*T*_1_	*p* value
Hb (g dL^−1^)	12.6 ± 2.2	12.1 ± 2.3	**0.0001**
Hct (%)	39.3 ± 7.2	37.7 ± 7.6	**0.001**
PLT (× 10^3^ µL^−1^)	217 ± 92	212 ± 89	0.65
BE (mmol L^−1^)	− 3.3 ± 3.9	− 3 ± 4.1	0.41
PTr	1.52 ± 2.15	1.53 ± 1.9	0.72
PTTr	1.03 ± 0.62	1.04 ± 0.59	0.78
INR	1.43 ± 1.75	1.44 ± 1.56	0.69

*p* values < 0.05 are in bold

*Hb* blood hemoglobin, *Hct* hematocrit, *PLT* blood platelets, *BE* base excess, *PTr* prothrombin time ratio, *PTTr* partial thromboplastin time ratio, *INR* international normalized ratio

TIC was found in 7 (9.7%) patients at *T*_0_ and in 9 (11.2%) patients at *T*_1_. Blood mean FBN levels resulted in 207 ± 66 mg dL^−1^ at *T*_0_ and 193 ± 72 mg dL^−1^ at *T*_1_. Blood fibrinogen had a statistically significant change from *T*_0_ to *T*_1_ (*p* = 0.02), but it was not detected as a statistically significant trend.

Hypoperfusion indices are shown in Table 2. The average amounts of fluids were: 779 ± 424 mL of crystalloids and 759 ± 365 mL of colloids; crystalloids and colloids were administered to 68 (81.9%) and 17 (20.5%) patients, respectively. A hypertonic solution, from 100 to 250 mL, was given to 8 (9.6%) patients. Blood products were administered in 35 (42.2%) patients within first 24 h after hospital admission: red blood cells (RBCs) were given in 31 (37.3%), fresh frozen plasma (FFP) in 16 (19.3%) and platelets in 4 (4.8%) patients. Twenty patients (24%) received tranexamic acid with no predefined criteria. The vital and laboratory parameters of this group showed no differences from patients who did not receive tranexamic acid.

Average TEG values at *T*_0_ and *T*_1_ are reported in Table 4. Stratification based on NISS showed no difference in TEG parameters between *T*_0_ and *T*_1_, except for statistically significant *R* value in both groups NISS 25–40 and NISS > 40 (*p* = 0.04 and *p* = 0.02, respectively). No correlation between TEG parameters and hypoperfusion parameters (sAP, BE, and StO_2_) or temperature was found. In the tranexamic acid group, TEG parameters were similar to others.

**Table 4:** TEG values at *T*_0_ and *T*_1_ (mean ± SD)

	*T*_0_	*T*_1_	*p *value
*R* (min)	7.54 ± 3.5	5.8 ± 3.1	0.10
*k* (min)	3.51 ± 3.6	2.87 ± 3.7	0.59
Angle (grades)	54.39 ± 14.1	60.38 ± 10.4	0.10
MA (mm)	57.87 ± 8.5	58.97 ± 10.4	0.50
*G* (dyne/s)	7.09 ± 2.3	7.71 ± 2.3	0.41

*R* *R*-time, *k* *K* value, *Angle* alpha angle, *MA* maximum amplitude

**Fig. 1:**
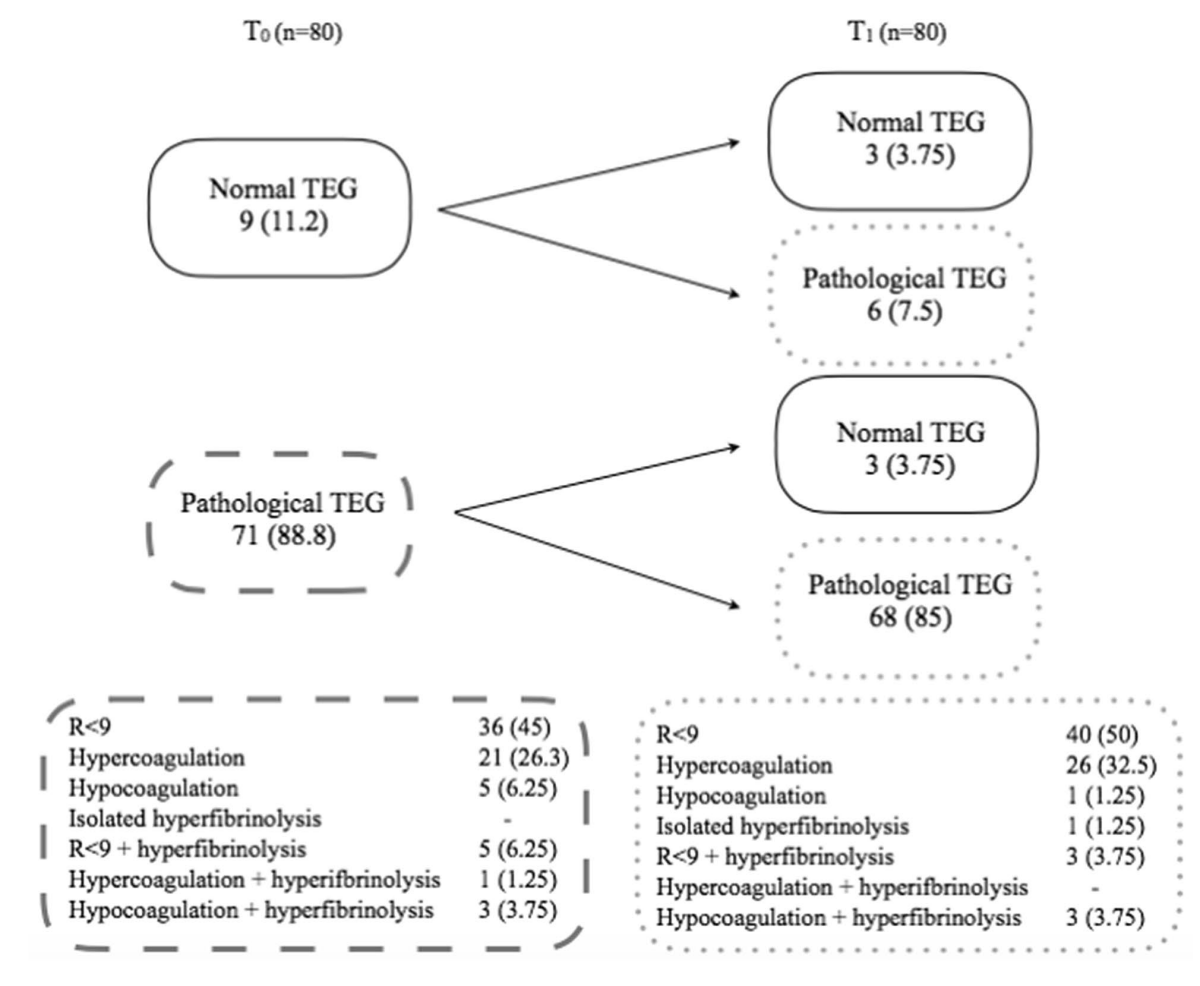
Changes in pathological and normal TEG from *T*_0_ to *T*_1_. And TEG features at *T*_0_ and *T*_1_. Data are presented as No. of patients and percentage

Nine patients had normal TEG pattern on the trauma scene, three of them maintained normal TEG while remaining 6 patients underwent different changes: 4 (5%) patients developed shortening of *R*, 1 (1.25%) showed hyperfibrinolysis, and 1 (1.25%) developed hypercoagulation (Fig. 1). Three (3.75%) pathological TEG turned to normal at *T*_1_. Among them, 2 (2.5%) patients showed isolated shortening of *R*, and 1 (1.25%) had hypercoagulation at *T*_0_.

The distribution of TEG features is summarized in Fig. 1 (three patients were lost for the coagulation analysis because TEG parameters were absent at *T*_0_ or *T*_1_). No difference in the prevalence of hypercoagulation and *R* < 9 patterns at *T*_0_ and *T*_1_ between NISS groups was detected. Within the same NISS group, there were no differences in the prevalence of hypercoagulation and *R* < 9 patterns between *T*_0_ and *T*_1_. In patients who received tranexamic acid, no difference in the prevalence of hyper or hypocoagulation patterns was detected compared to the patients that did not receive tranexamic acid.

About changes in TEG features from *T*_0_ to *T*_1_: 17 TEG worsened, mostly (8 patients, 10%) becoming hypercoagulant from an isolated shortening of *R*, while 19 improved, mainly (12 patients, 15%) acquiring isolated shortening of *R* from several patterns (hyper, hypocoagulation or hyperfibrinolysis). Forty-four patients had no changes in TEG features, maintaining chiefly activation of coagulation (*R* < 9 in 23, 28.7%, and hypercoagulation in 15, 18.8%, patients). (Table S1 on Supplemental Material).

Among patients with TIC, 3 had a normal TEG, 3 had hypercoagulation, and one had isolated shortening of *R* at *T*_0_. While at *T*_1_, 3 patients had normal TEG, 2 had hypercoagulation, 2 had hypocoagulation, and 2 had isolated shortenings of *R*.

We divided our study population according to changes in the TEG pattern (worsened vs. not worsened). Searching for the different prevalence of hypoperfusion parameters on the scene and at hospital admission (Table 5), only StO_2_ < 75% at *T*_0_ showed a statistically significant difference in its prevalence in two groups (*p* = 0.02). No difference was found between the groups for the amount of fluid administrated. Worsened TEG group showed a mean temperature of 35.2 ± 3 °C, while the other group had 36.2 ± 0.7 °C, with no statistically significant difference between *T*_0_ and *T*_1_.

**Table 5:** Hypoperfusion parameters and fluid therapy (No. of patients and percentage) between two groups based on TEG features (worsened vs. not worsened)

	Worsened (*n* = 17)	Not worsened (*n* = 63)	*p* value	Worsened (*n* = 17)	Not worsened (*n* = 63)	*p* value
Patients with	*T*_0_			*T*_1_		
sAP < 90 mmHg	4 (23.5)	8 (13.8)	0.33	2 (11.8)	2 (3.4)	0.18
sAP ≥ 90 mmHg	13 (76.5)	50 (86.2)		15 (88.2)	56 (96.5)	
BE ≤ − 6 mmol L^−1^	2 (15.4)	10 (18.2)	0.8	4 (25)	10 (18.2)	0.54
BE ≥ − 6 mmol L^−1^	11 (84.6)	45 (81.8)		12 (75)	45 (81.8)	
StO_2_ < 75%	7 (46.7)	8 (17.4)	**0.02**	1 (7.7)	5 (11.1)	0.72
StO_2_ ≥ 75%	8 (53.3)	38 (82.6)		12 (92.3)	40 (88.9)	

Fluids	Worsened	*p* value
≥ 1000 mL (1454 ± 685)	6 (35.3)	0.72
< 1000 mL (478 ± 113)	11 (64.7)	

*p* values < 0.05 are in bold

Amounts of fluid administered are reported in brackets as mean ± SD

*sAP* systolic arterial pressure, *BE* base excess, *StO*_*2*_ tissue oxygen saturation

## Discussion

### Early alteration of coagulation and hyperfibrinolysis

In our study, we found almost all (88.8%) trauma patients had a pathologic TEG already on trauma scene. The most represented pathological TEG patterns were shortening of *R* or hypercoagulation. These findings suggest that strong activation of the hemostatic system occurs within 30 min from trauma, and is maintained at hospital arrival.

Kaufmann et al. found that at hospital admission, the most represented hemostatic feature in an adult trauma population was hypercoagulation (included isolated shortening of *R*) [19]. Other TEG-versus-ROTEM comparison studies described a reduction in mean *R* value on early post-hospital admission TEG as well [20, 21].

In single TEG parameters analysis, the only *R* mean value showed a significant difference between on-scene and hospital arrival: in a large majority of patients was detected an *R* value decreasing.

Only another study searched for early post-traumatic coagulation abnormalities with TEG. This study was similar to ours, but our population presented more severe injuries [17]. The authors found no differences in TEG values between on-scene and hospital admission, except for a slight increase in MA, whose mean value remains in a normal range. In this study, *R* mean value at hospital admission was at the lower limit of the normal range. This finding could be due to a smaller magnitude of trauma, so the activation of coagulation was still setting up.

Almost one-fourth of patients had a worsening in TEG pattern from “on the scene” to hospital arrival. Despite this finding, there was no significant difference in standard coagulation parameters between the on-scene and hospital admission setting. This observation could be due to the time frame we chose for analysis. In our data, a worsening in TEG was mostly represented by a hypercoagulation pattern.

Gonzalez et al. suggested that *R* value could be analogous to PT and PTT. These latter values represent the activation of coagulation factors, even if they are not interchangeable to each other [22]. In literature, *R* value showed a strong correlation with PT and aPTT [23]. Despite significant changes in *R* values between the two groups which we had detected, we found no difference in variations of PT and PTT.

Very early after trauma, coagulation modifications are expected to be towards activation, so we aspected a shortening of *R*. Moreover, according to pathophysiology, changes in PT and PTT become substantial later in the clinical course of the traumatized patient. When coagulation starts failing, a hypocoagulation pattern could be detected (which represents the alteration of coagulation for which these laboratory tests were specifically designed). Standard laboratory assays were developed to diagnose hypocoagulant conditions predominantly, so their application in very early post-traumatic settings may hide the gain of a hypercoagulant profile.

Hyperfibrinolysis was found in a few patients (11.3%) on the scene, and its prevalence showed a slight decrease at hospital admission (8.8%). Our prevalence of hyperfibrinolysis at hospital admission is smaller than reported by the literature [24, 25] using the same Ly30 cut-off. It may be due to differences in trauma patients, who are mostly hemorrhagic, requiring massive transfusions protocol in Chapman et al. [24]. Moore et al. enrolled more patients than us, so it could maybe affect the sensitivity of detection. We did not find an increase in hyperfibrinolysis from the trauma scene to hospital admission. Trauma patients are at increased risk of developing shock-induced hyperfibrinolysis due to pathological endothelial activation secondary to hypoperfusion and sympathoadrenal activation [26]. A few patients in our population presented more than two hypoperfusion markers on the trauma scene. The strategies to correct the hypoperfusion in the pre-hospital environment could probably correct the coagulation factors.

A small number of patients in our population developed TIC: 9.7% on the scene and 11.2% at hospital admission. As there is not a unique definition of TIC, different prevalences are reported depending on the cut-off value used. In 2003 Brohi et al. [4] published a large retrospective study, reporting that 24.4% of trauma patients had trauma acute coagulopathy (defined as PT > 18, aPTT > 60 s or TT > 15 s) at hospital admission. More recently, Davenport et al. found that 8% of trauma patients had PT ratio > 1.2. At the same time, Tauber et al. [16] and Hagemo et al. [15] using two different INR cut-off (1.5 and 1.2, respectively), found a coagulopathy in 14% and 11% of patients. In particular, Tauber et al. presented a population very similar to ours.

Patients with TIC did not exhibit a specific TEG pattern. They showed all possible kind of coagulation response to trauma: from normal to hyper- and hypocoagulant profile, to isolated shortening of *R* value. While prolonged INR and hypocoagulation move in the same direction, the finding of prolonged INR with normal or hypercoagulation pattern seems inconsistent. Standard laboratory tests detect the earliest initiation of clot formation [27], and PT/INR are extremely sensitive to clotting factors II, VII, and X [28]. Viscoelastic methods provide information about the whole coagulation process, exploring the interaction between all different components of the hemostatic system [29].

According to the cell-based paradigm of coagulation, TIC involved two critical players: platelets and endothelial cells. Immediately after trauma, the exposition of tissue factor from damaged tissues initiates coagulation through factor VII activation (the so-called “extrinsic pathway”). Factor VII is present in very low concentration in plasma and has the shortest half-life among all other coagulation factors [30]. Amplification and propagation are consequences of activation of endothelial cells, platelets, and other coagulation factors [27], also from the so-called “intrinsic pathway” [31]. The concomitance of two conditions may explain a prolonged INR in the presence of a hypercoagulation pattern. The initiation of coagulation determines the consumption of circulating factors to which INR measurement is sensitive. The amplification and propagation of coagulation strengthen the clot, whose “strength” is represented only by VHAs.

Our study was not designed to search for a correlation between laboratory tests and VHA in the diagnosis of TIC. Despite this, our findings suggest that even if the on-going activation of coagulation, conventional coagulation tests could be normal during early blood loss [14]. Furthermore, standard laboratory tests may suggest normal coagulation in patients with hypercoagulation pattern at VHA.

On the other hand we have to notice that our results showed a little number of patients with INR > 1.5 and normal TEG (and thus normal *R*). This sounds like a surprising discrepancy between PT/INR and *R* time. In a recent study of Sumislawski, the authors found that 20% of patients with abnormal INR have normal ACT on rapid-TEG (which is a substitute of *R* time of TEG); moreover, the case studies of these authors were more severely injured and had lower activity levels for all coagulation factors (except for VIII) respect to patients with isolated abnormal ACT. Severity of injury and factor II level were identified by authors as independent predictors of discordance between conventional and viscoelastic test.[32]. However, there is not a still clear explanation of this discrepancy but, as Sumislawki suggests, it may reflect differences in coagulation factor activities and thus different clinical phenotypes.

### TEG and NISS

The different coagulation patterns which we detected did not show any correlation with NISS value at any time. Regardless of when the determination is made, in the case of severe trauma, many patients show a hypercoagulation profile. No difference in *R* values from the scene to the hospital admission for NISS 15–24 was detected. In NISS 25–40 patients, a moderate difference was found; the difference was very significant for NISS > 40 patients. In other words, the development of a hypercoagulation pattern is due to the severe trauma itself. More severe it is injury, greater is the magnitude of coagulation activation (*R* shortening).

### TEG and hypoperfusion indices

Systolic arterial pressure and BE had not different distribution between patients whose TEG worsened and patients whose TEG remained stable or improved. At the same time, StO_2_ < 75% was more present in the worsened TEG group. Thus, clinical features may not help to identify patients with ongoing post-traumatic hemostatic deranging early. There are a few studies that found a correlation between a hypoperfusion parameter such as BE, alone or with other clinical and laboratory features, and a single coagulation factor like blood fibrinogen [33, 34], but no one used VHA.

### TEG and fluids

Hypercoagulation pattern at hospital admission was less represented in the group of patients who received more than 1000 mL of fluid for resuscitation than in the group of patients who received 1000 mL or less. No other TEG pattern presented different distribution between the two groups depending on the amount of fluid administration. Fluid therapy may have affected, through hemodilution, the real prevalence of hypercoagulant status at hospital admission. 10% of our patients presented hypocoagulation, hyperfibrinolysis, or a combination of the two patterns, on the scene. Furthermore, despite a liberal fluid administration, at hospital admission, these patterns were less represented (6%).

The fluid administration could not be sufficient to cause a dilutional coagulopathy. Furthermore, hemodilution alone is known to be not enough to set up coagulopathy [27]. Post-traumatic coagulation changes are expected to be pro-coagulant, at least in the very first phase [35]. If hypoperfusion is not counteracted, hypocoagulation develops mediated by thrombomodulin via activated protein C [36], and, at last, hypercoagulation takes place again, leading to thrombotic complications [37]. In our population, the first samples were collected very early, so it is not surprising to find mostly hypercoagulation pattern. However, the second samples were collected in a time frame in which we could have expected a rising number of hypocoagulant profile. This second event did not take place because of blood loss, and resulting hypoperfusion was probably counteracted with the pre-hospital fluid administration. This management could interrupt the pathway that would have led to hypocoagulation.

### TEG and temperature

Most of our patients had only mild hypothermia on the scene, and at hospital admission as well. Mild hypothermia does not reduce enzyme activity [38, 39] and seems not to affect platelet activation. It could only cause platelet adhesion defects [38]. Moreover, hypothermia plays a role in coagulation alteration later in the clinical course of traumatized patients [36]. In our population, temperature alteration was too small to take part in early hemostatic derangement.

## Limitations

During data collection, there were changes in fluid therapy from liberal to restrictive. Thus fluid administration in our population is not similar to the more recent literature on trauma. Furthermore, some patients received moderate volumes of colloids that are thought to impair coagulation. Our study population had a wide distribution of age, NISS, mechanism, and type of injury. Injuries were mostly thoracic and cranial, and only one-third of patients needed a blood transfusion during the first 48 h after trauma. On average, patients maintained hemodynamic stability. They also had a good balance of laboratory parameters such as hemoglobin, platelets, and base excess. No differences were observed between on-scene and hospital admission, according to almost all hypoperfusion indices. Thus our population is not so representative of major bleeding trauma but more likely of a non-hemorrhagic subgroup of severe injury.

## Conclusion

Our study shows that trauma-related alterations in coagulation are early on the trauma scene. The shortening of *R* and a hypercoagulation profile are the most represented coagulation alterations. Hyperfibrinolysis is found in a low percentage of patients, probably due to the non-hemorrhagic nature of our trauma population. Pro-coagulant changes develop very early after trauma, regardless of the severity of the injury. Damage severity appears to affect only the *R* parameter on TEG determination. Hypoperfusion parameters do not help to identify patients with ongoing coagulation impairment. In non-hemorrhagic severe trauma patients, small volume resuscitation does not affect changes in the coagulation pattern at VHA. Mild hypothermia does not affect coagulation, at least in the early post-traumatic phase.

## Electronic supplementary material

Below is the link to the electronic supplementary material.

Supplementary file1 (DOC 44 kb)
