# Supplementary material for: Early thromboelastography in acute traumatic coagulopathy: an observational study focusing on pre-hospital trauma care
Source: Eur J Trauma Emerg Surg. 2020 Sep 14;48(1):431–9. doi: 10.1007/s00068-020-01493-z (PMC8825617; doi:10.1007/s00068-020-01493-z)
Supplement: Supplementary file 1 — Supplementary file1 (DOC 44 kb) [file 68_2020_1493_MOESM1_ESM.doc]

| TEG features from T0 to T1 | N° pts (%) |
| --- | --- |
| Stable (n=44) | |
| Normal | 3 (3.75) |
| R<9 | 23 (28.7) |
| Hypercoagulation | 15 (18.8) |
| Hypocoagulation + hyperfibrinolysis | 2 (2.5) |
| R<9 + hyperfibrinolysis | 1 (1.25) |
| Worsened (n=17) | |
| R<9 → hypercoagulation | 8 (10) |
| R<9 → hypocoagulation | 1 (1.25) |
| R<9 → R<9 + hyperfibrinolysis | 1 (1.25) |
| Normal → R<9 | 4 (5) |
| Normal → hypercoagulation | 1 (1.25) |
| Normal → + hyperfibrinolysis | 1 (1.25) |
| Hypocoagulation → R<9 + fibrinolysis | 1 (1.25) |
| Improved (n=19) | |
| Hypercoagulation → R<9 | 5 (6.25) |
| Hypocoagulation → R<9 | 4 (5) |
| R<9 + hyperfibrinolysis → R<9 | 3 (3.75) |
| R<9 + hyperfibrinolysis → hypercoagulation | 2 (2.5) |
| R<9 → normal | 2 (2.5) |
| Hypercoagulation → normal | 1 (1.25) |
| Hypercoagulation + hyperfibrinolysis → hypercoagulation | 1 (1.25) |
| Hypocoagulation + hyperfibrinolysis → R<9 | 1 (1.25) |

*Table S1. Changes in TEG features (N° of patients and percentage) from T0 to T1. Legend: R = R-time.*
